# Implementation of Hospital-to-Home Model for Nutritional Nursing Management of Patients with Chronic Kidney Disease Using Artificial Intelligence Algorithm Combined with CT Internet +

**DOI:** 10.1155/2022/1183988

**Published:** 2022-03-27

**Authors:** Xing Chen, Xueqin Huang, Mingyuan Yin

**Affiliations:** ^1^Department of Internal Medicine, Hunan Provincial People's Hospital, Changsha 410005, Hunan, China; ^2^Department of Nursing, Hunan Provincial People's Hospital, Changsha 410005, Hunan, China

## Abstract

The objective of this study was to evaluate the application value of “Internet + hospital-to-home (H2H)” nutritional care model using the improved wavelet transform algorithm based on computed tomography (CT) images in the nutritional care management of chronic kidney disease (CKD) stages 3-5. A total of 120 patients with CKD were the research objects and they were randomly divided into two groups. The normal nutritional nursing model was used for nursing of patients in the control group, and the “Internet + *H*2H″ model was used for the observation group (H2H group), with 60 cases in each group. The nursing effect was evaluated using 320-slice volume CT low-dose perfusion imaging images, anthropometry, laboratory biochemical tests, and other survey scores. The results showed that compared with the mean filter denoising (MFD) algorithm and the orthogonal wavelet denoising (OWD) algorithm, the mean square error (MSE) and signal noise ratio (SNR) values of the IWT algorithm were better (40.0781 vs 45.2891, 59.2123)/(20.0122 vs 18.2311, 15.7812) (*P* < 0.05). The arm muscle circumference (MAC) (239.77 ± 18.24 vs 243.94 ± 18.72 mm) and triceps skindold (TSF) value (8.87 ± 2.74 vs 10.04 ± 2.90 mm) of the patients in the H2H group were greatly improved after the nursing (*P* < 0.05). For biochemical indicators, serum albumin (ALB) (35.22 ± 4.98 vs 45.32 ± 4.21) g/L, prealbumin (PAB) (289.94 ± 72.99 vs 341.79 ± 74.45) mg/L, hemoglobin (Hb) (97.62 ± 24.87 vs 110.65 ± 28.83) g/L, and blood urea nitrogen (BUN) (15.74 ± 9.87 vs 11.06 ± 5.69) mmol/L of patients in H2H group were improved (*P* < 0.05). After nursing, the nutritional screening score of the H2H group was obviously improved (83.33% (before) vs 50% (after)), the total score of health quality assessment (114.89 ± 5.23) in the H2H group was much higher than that of the control group (87.22 ± 14.89), and the satisfaction on the nursing model was higher in the H2H group (100% vs 71.67%) (*P* < 0.05). The renal cortex BF before and after nursing was significantly different between the two groups of patients (*P* < 0.05), and the BE of the H2H group was significantly higher than that of the control group after treatment ((335.12 ± 52.74) mL·100 g^−1^·min^−1^ vs (289.90 ± 53.91) mL·100 g^−1^·min^−1^) (*P* < 0.05). In summary, the “Internet + *H*2H″ nutritional nursing model was more individualized, which can better improve the physical quality of patients with stages 3-5 of CKD, improve the psychological state of patients, and further enhance the prognosis of the disease. In addition, the IWT algorithm showed better effects in the processing of the image of 320-slice volume CT low-dose perfusion imaging, and it was worthy of clinical application.

## 1. Introduction

Chronic kidney disease (CKD) refers to the structure and function of the kidney caused by various reasons, and the onset time is more than 3 months [1, 2]. With the continuous increase in the number of CKD patients in recent years, the number of CKD patients worldwide has exceeded 850 million, and China has also exceeded 100 million [3]. It has been clinically found that patients with CKD 3-5 stages are prone to abnormal gastrointestinal function, endocrine dysfunction, metabolic acidosis, comorbidity [4], systemic microinflammatory state [5], decreased renal function, and accumulation of uremic toxins, causing a series of problems in the patient's body nutrition [6]. According to statistics, the disability rate and fatality rate of CKD rank first in chronic diseases, which is greatly related to the nutritional problems in the development of CKD [7]. Therefore, the improvement of nutritional problems is of great significance for improving the quality of life of patients and reducing the mortality and disability rate of patients.

At present, the commonly used clinical nutritional management method for chronic diseases is a continuous nutritional nursing model, which is called hospital to home (H2H). It is a hierarchical management model of hospital-community-home, emphasizing the continuity, individualization, and participation of patients and family members of nutritional management [8]. Studies have shown that the continuous and professional H2H management model meets the needs of patients in many ways and improves the overall effect of treatment [9]. In recent years, Internet technology has also been integrated into the H2H nutritional nursing model, forming an “Internet + *H*2H″ model. “Internet +” can use information technology and Internet platforms to deeply integrate various fields of society, so as to optimize the allocation and integration of social resources and create a new development ecology [10]. Studies have shown that a service platform based on “Internet +” can retain all nursing resources, which is convenient for patients to check repeatedly and enhance self-nursing awareness [11].

To further understand the application effect of the “Internet +” H2H nutrition care model in patients with CKD, CT was adopted to evaluate the renal status of the patients before and after the application of the nutrition care model. Clinical studies have shown that the blood flow status in the kidney can reflect its physiological and pathological conditions, and CT perfusion imaging of the kidney is an examination method that shows the microcirculation information of living tissues [12, 13]. At this stage, the radiation and the reproducibility of perfusion results in renal CT perfusion imaging technology are clinical research hotspots. The study proposed a 320-slice CT 16 cm wide detector. The scanning mode used by 320-slice CT is the nonmoving bed volume perfusion mode. The kidneys are in the same phase during the scanning process, which makes the measurement of renal cortex blood flow more accurate [14]. In order to reduce the amount of radiation in the CT examination process, low-dose perfusion can also affect people's perfusion. However, most of the images in the low-dose imaging process will have noise pollution, and the image display is not clear, which affects the accuracy of the examination results. In order to make the results of CT perfusion imaging more conducive to analysis and comparison, artificial intelligence algorithms were adopted in this study to denoise CT images. The wavelet transform denoising algorithm has been extensively studied in the optimization of CT images [15]. In order to improve their denoising performance, some research experts have carried out improved research on the wavelet transform algorithm. Research has shown that the improved wavelet transform denoising (IWT) algorithm can obtain effective application effects [16, 17].

In summary, this study used the Internet + *H*2H nutritional nursing model for patients with CKD in stages 3-5, adopted the 320-slice volume CT low-dose perfusion imaging examination image based on the IWT algorithm and other examination indexes for analysis and comparison, and evaluated the application value of the “Internet + *H*2H″ nutritional nursing model, aiming to provide a reasonable research basis for continuous nutrition management services for CKD patients in clinical practice.

## 2. Research Methods

### 2.1. Research Objects

In this study, 120 patients with CKD admitted to the hospital from March 2019 to March 2021 were randomly selected as the research objects. Among them, 66 were male patients and 56 were female patients. They were between 38 and 78 years old, with an average age of 62.34 ± 13.09 years old. There were 4 patients with an education level of elementary school and below, 34 with junior high school, 64 with high school/technical secondary school, and 18 with junior college and above. There were 25 patients with a family monthly income of less than RMB 3,000 and 95 patients with RMB 3,000 and above; there were 32 patients with CKD in stage 3, 58 patients with stage 4, and 40 patients with stage 5. A random number table method was used to divide 120 patients into two groups, which were set as the control group and the observation group (H2H group). The number of patients in both groups was 60. The patients in the control group used the conventional nutritional nursing model for nursing, and the patients in the H2H group use the “Internet +” H2H nutritional nursing model. Then the 320-slice volume CT low-dose perfusion imaging images based on the IWT algorithm, anthropometry, laboratory biochemical testing, and other survey scores were adopted to evaluate the application effect of the nutritional nursing model of the two groups of patients. This study had been approved by the ethics committee of hospital and all the research objects included in this study had signed the agreement.

Patients included in this study had to meet the following criteria: patients who met the diagnostic criteria of CKD [18], patients with CKD in stages 3-5 (the clinical staging criteria of CKD are listed in Table 1) and in stable condition. Patients whose main caregivers supported cooking at home or eating out <3 times/week, patients with clear consciousness and with certain language skills and smartphones, patients ≥18 years old, and patients who signed the informed consents and voluntarily participated in this study.

Patients who met the following criteria had to be excluded from this study: those with severe cognitive, mental, and language impairment; those with severe complications and severe damage to important organs; patients who received renal replacement therapy; those who were participating in other intervention studies.

### 2.2. Interventions

Patients in the control group received the conventional nutritional nursing model, while the patients in the H2H group were received the “Internet+” combined H2H management model.

#### 2.2.1. Routine Nutrition Management Model

- Routine health education after admission: to inform patients and their families about the importance and continuity of nutritional support for CKD and the necessity of standardized treatment and follow-up outside the hospital.
- To choose a variety of foods that are nutritionally sound: it should be emphasized that adequate energy intake should be ensured while appropriately restricting protein intake to prevent malnutrition.
- Regular and quantitative meals: the energy of breakfast, lunch, and dinner can account for 20%–30% and 30%–35% of the total energy. The protein of three meals should be evenly distributed.
- To limit the intake of plant proteins such as rice and noodles: it can take wheat starch (or other starches) as the staple food to replace ordinary rice and noodles and add appropriate amounts of milk, eggs, or various meats, soybeans, and other high-quality protein foods.
- When the disease requires restricting foods with high phosphorus content, it should carefully choose animal liver, nuts, dried beans, and various phosphorus-containing processed foods.
- When the disease requires restricting foods with high potassium content, it should carefully choose fruits, potatoes and their starches, green leafy vegetables, etc.
- Discharge guidance before discharge should be guaranteed with high quality, and regular telephone follow-up must be carried out after discharge to instruct patients to follow up on time.

#### 2.2.2. Internet + H2H Care Model

First of all, the H2H management team and wechat management team should be established. The team consisted of 1 team leader (as the head nurse of the section), 1 consultant (as the section director), and 6 team members (1 nephrologist, 5 specialist nurses, and 1 dietitian). Secondly, all members of the team had to be trained. The training contents included kidney disease nutrition-related knowledge (9 credit hours), Internet nutrition management platform operation and common problem handling (1 credit hour), enrollment and data collection of patients (1 credit hour), and communication skills (1 credit hour).

The nephrologist was responsible for the overall treatment plan. The nutritionist was responsible for formulating individualized nutrition support plans and data collection for patients during and after discharge from the hospital and recorded clinical data of inpatients-including general data (name, gender, age, height, weight, blood pressure, blood sugar, etc.) and laboratory indicators (blood creatinine, urea nitrogen, uric acid, electrolytes, etc.) on the Internet platform. Specialist nurses were responsible for guiding patients to register on the Internet platform, providing platform operation guidance for patients and main caregivers (general data and laboratory index entry-data entry or uploading photos, online consultation, recipe viewing, etc.), ensuring that patients and the main caregiver were proficient in platform operation and other issues such as health education, data collection, assistance, and supervision of the implementation of the plan. Group members communicated and discussed the patient's situation once a month through video conferences and kept in close contact through WeChat groups during the rest of the period. After the initial treatment plan and nutrition plan of the patient were formulated, it was necessary to study and discuss it in the WeChat group to ensure that all members of the management team were fully aware and understood and to discuss the feasibility of the plan and the cooperation steps among members.

After the patient was admitted to the hospital, the nutritionist formulated individualized dietary prescriptions based on the patient's general information, laboratory indicators, allergy history, and communication with the nephrologist, explained the dietary prescriptions to the patient and the main caregivers in detail, and informed the precautions. The patient's general information and laboratory indicators were entered into the Internet platform. The platform automatically generated recipes for three meals a day, and the recipes were updated every three days. Group members patiently answered questions raised by patients.

On the day, the patient was discharged from the hospital, the specialist nurse urged the patient and the main caregiver to enter the patient's last laboratory indicators and the patient's weight, blood pressure, blood glucose, and other data into the Internet platform and checked whether the data entry was correct. After the patients discharged from the hospital, the Internet platform automatically sent three meals a day recipes and health education to the patients, reminded the patients to return to the clinic, and provided online consultation services to the patients. During the stay at home, patients should go to the community or hospital for re-examination according to the platform's re-examination reminder time limit and upload the re-examination laboratory indicators and general information to the Internet platform in time. Nutritionists assessed the nutritional status and support needs of the patients based on the results of the patient review and screened patients with malnutrition and high risk. Specialist nurses supervised and urged patients to implement nutritional programs. If patients had any questions, they can consult a doctor, nutritionist, or specialist nurse online on the platform.

### 2.3. IWT Algorithm

Among the wavelet transform algorithms, the image denoising method of OWD [19] has been widely used in image processing. However, this method can produce artificial noise, and the image reconstruction can affect a single wavelet coefficient due to the degree of translation. It has a strong dependence, which makes it difficult to choose the wavelet denoising threshold. Research shows that the dyadic wavelet transform (DWT) algorithm has translation invariance. Therefore, under the same misjudgment probability, the DWT algorithm has a better denoising effect [20].

In the processing of two-dimensional images, the DWT algorithm used multiple mother wavelet functions in different spatial directions for calculation, which could be specifically expressed as follows:

(1) $$\begin{array}{c} {\left\{\varphi^{d}\right\}}_{1\leq d\leq D}. \end{array}$$

Here, *d* represented the airspace direction, *d* *=* *1* represented the horizontal direction, *d* *=* *1* represented the vertical direction, and *φ* referred to the wavelet function. The two-dimensional discrete function can be expressed as follows:

(2) $$\begin{array}{c} f\left(m,n\right)\in l^{2}\left(z^{2}\right). \end{array}$$

Here, *l* represented the parameter in the horizontal direction and *z* represented the dispersion coefficient. Then, the corresponding finite-order dyadic wavelet transform can be expressed as follows:

(3) $$\begin{array}{c} W\left[f\left(m,n\right)\right]=\left\{W_{j}^{d}{\left[f\left(m,n\right)\right]}_{d=1,2;1\leq j\leq J},S_{J}\left[f\left(m,n\right)\right]\right\}. \end{array}$$

Here, *(m, n)* represented the position, *j* represented the scale, *W*_*j*_^*d*^[*f*(*m*, *n*)] referred to the transform coefficient of the wavelet function *f*(*m*, *n*) at the location of *(m, n)*, scale of *j*, and direction of *d*, and *S*_*J*_[*f*(*m*, *n*)] was the approximate value of the location of *(m, n)* and the maximum scale of *J*.

In the denoising process of the DWT algorithm, the selection of the threshold is a key issue. The general threshold is easy to smooth the image, making the image too blurry. In this study, a new threshold was adopted, which could be calculated with the following equation:

(4) $$\begin{array}{c} \underset{j}{\lambda }=\sqrt{\log\frac{L_{j}}{J}\frac{{\underset{j}{\sigma }}^{2}}{\beta_{j}}}. \end{array}$$

Here, *J* represented the number of decomposition layers of the scale, *L*_*j*_ represented the subband width on the j-th scale, $\underset{j}{\sigma }$ represented the standard deviation of the noise on the j-th scale, and *β*_*j*_ was the variance of the coefficient component matrix on each scale.

From the decomposition structure of the wavelet transform, the noise variance expression of the j-th order can be obtained as follows:

(5) $$\begin{array}{c} {\underset{j}{\sigma }}^{2}=\sigma^{2}{\left|\left(\sum_{i=0}^{j-2}*h_{i}\right)*g^{j-1}\right|}_{F}•{\left|\left(\sum_{i=0}^{j-2}*h_{i}\right)\right|}_{F}. \end{array}$$

Here, *σ*^2^ represented the variance of the original image noise, *∗* represented the convolution, ‖ _*F*_•_*F*_‖ represented the norm, *h*_*i*_ referred to the filter coefficient formed by inserting 2^*j*^ − 1 zeros after the filter coefficient *h*_*i*−1_, and *g*^*j*−1^ represented the filter coefficient *g*^*j*−2^ formed by inserting 2^*j*^ − 1 zeros before the filter coefficient. Since the variance of the noise of the original CT image was unknown, it needed to be estimated. If the wavelet coefficients on the smallest scale are used to estimate, the estimated value is as follows:

(6) $$\begin{array}{c} \sigma =\frac{\text{median}\left(\left|W_{j}^{d}f\right|\right)}{0.6745}. \end{array}$$

Since there was a certain correlation between the wavelet coefficients, in order to prevent the wavelet coefficients from being incorrectly set due to the correlation, the window was set. The window was centered on the wavelet coefficient *x*_*j*_, *k* that needed to be processed at the time, and the size was *m* × *m*{*m* > 1, |*m* ∈ 2*n*+1|*n* ≥ 0}, (2*n*+1 was the representation method of an odd number) was recorded as *C*_*m*_(*x*_*j*_, *k*), the maximum value *M*_*j*_ _,*k*_ of the absolute value of all wavelet coefficients in the current window is calculated, which can be expressed as follows:

(7) $$\begin{array}{c} M_{j}{\;}_{,k}=\max{\left|x_{m,n}\right|}_{\left(m,n\right)\in C_{m}\left(x_{j},k\right)}. \end{array}$$

Then, the following equation could be obtained according to equation (4):

(8) $$\begin{array}{c} x_{j},k=x_{j},k,\left|x_{j},k\right|\geq \lambda ,\\ x_{j},k=x_{j},k\left(1-\frac{\lambda -\left|x_{j},k\right|}{M_{j}{\;}_{,k}-\left|x_{j},k\right|}\right),\left|x_{j},k\right|<\lambda \&M_{j}{\;}_{,k}\geq \lambda ,\\ x_{j},k=0,\left(\text{others}\right). \end{array}$$

The specific denoising effect was evaluated by mean square error (MSE) and signal-to-noise ratio (SNR). The specific calculation method was as follows:

(9) $$\begin{array}{c} \text{MSE}=\frac{1}{M•N}\sum_{m=1,n=1}^{M,N}{\left(f_{m,n}-{\overset{⟶}{f}}_{m,n}\right)}^{2}. \end{array}$$

(10) $$\begin{array}{c} \text{SNR}=101g\left[\frac{\sum_{m=1,n=1}^{M,N}f_{m,n}^{2}}{\sum_{m=1,n=1}^{M,N}{\left(f_{m,n}-{\overset{⟶}{f}}_{m,n}\right)}^{2}}\right]. \end{array}$$

Here, *f*_*m*,*n*_ represented the original image, ${\overset{⟶}{f}}_{m,n}$ represented the image after denoising, and *M*•*N* represented the size of the image.

### 2.4. CT Examination

**Figure 1:**
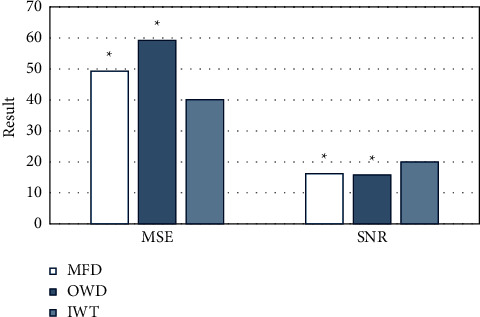
Comparison on MSE and SNR of different algorithms. *∗P* < 0.05 indicates the difference was statistically significant, compared with “IWT algorithm.”

The 320-row volumetric CT was used for inspection in this study. Before scanning, the patient was required to lie on their back and relax their breathing, and the patient was bound with an abdominal band to fix it to reduce the range of activity. Firstly, the positioning image was adopted to scan and locate the positions of both kidneys, so that the effective width of the detector (maximum 16 cm) included the entire kidneys on both sides. Secondly, it should perform dynamic volume perfusion enhanced scanning. The specific operation is shown in Figure 1. The scanning parameters were given as follows: tube voltage was −120 kV, tube current was −55 m As, rotation time was −0.6 S, collimation was −0.5 mm × 300, layer thickness was 0.6 mm, repetition time was 4 s, number of times was 15 times, and total time was 60 s. The examination table should not be moved during scanning.

### 2.5. Data Processing

The image was transferred to the vetra postprocessing workstation, and the scan information of the image was corrected and analyzed by the BODY RESITATION system. The region of interest (ROI) was set. According to the imaging characteristics, 2 ROIs were set in the renal cortex at the renal upper pole, lower renal pole, and renal hilum level. The time-density curve (TDC) of the ROI was drawn, and a perfusion function map that can reflect the perfusion state of the renal blood flow was obtained, which referred to the renal blood flow (BF) map.

### 2.6. Evaluation Tools

Anthropometric indicators were used for evaluation, so the triceps skindold (TSF) and arm muscle circumference (MAC) of the two groups of patients were measured and compared before nutritional nursing and 6 months after nursing.

It should test and compare the laboratory biochemical indicators of the two groups of patients before nutritional nursing and 6 months after nursing, including serum albumin (ALB), prealbumin (PAB), hemoglobin (Hb), blood urea nitrogen (BUN), and serum creatinine (SCr).

Nutrition risk screening 2002 (NRS 2002) [21] (total score ≥3 points to indicate that the patient has malnutrition or nutritional risk) and health survey summary (the MOS item short from health survey, SF-36) [22] were adopted. Satisfaction questionnaires were adopted to evaluate and compare the physical condition, quality of life, and nursing satisfaction of patients before and 6 months after nutritional nursing.

The 320-slice volume CT low-dose perfusion images based on improved wavelet transform denoising algorithm were selected in this study to examine the changes of renal cortex SF before nutritional nursing and 6 months after the nursing. Then, the renal recovery of the two groups of patients was compared.

### 2.7. Statistical Analysis

SPSS 20.0 statistical software was used to analyze the data. The count data were expressed as n/%, and *χ*2 test was used. The measurement data were expressed as ‾*x* ± *s*, and the *t*-test was used. *P* < 0.05 indicates the difference was statistically significant.

## 3. Results

### 3.1. Comparison on General Data of Patients

The general clinical data of the two groups of patients were compared. In the control group, there were 34 males (51.52%) and 27 females (48.21%); there were 32 males (48.48%) and 29 females (51.79%) in the H2H group. The average age of patients in the control group was 61.72 ± 13.69 years, and that in the H2H group was 63.92 ± 13.29 years. In the control group, there were 2 patients with education level of elementary school and below, 18 with junior high school, 33 with high school/technical secondary school, and 10 with junior college or above. In the H2H group, there were 2 patients with primary school education and below, 16 cases with junior high school, 31 cases with high school/secondary school, and 8 cases with junior high school and above. In the control group, the number of patients with per capita monthly income of less than 3,000 yuan was 13 cases and 47 cases were those with 3,000 yuan and above; while those in the H2H group were 12 cases and 48 cases. In the control group, there were 15 patients in stage 3, 28 patients in stage 4, and 19 patients in stage 5; while in the H2H group, there were 17 patients in stage 3, 30 patients in stage 4, and 21 patients in stage 5. After analysis and comparison, the comparison of the general clinical data distribution between the two groups of patients was not statistically significant (*P* < 0.05), suggesting the feasibility of the comparison of this study.

### 3.2. Comparison on Performance of Denoising Algorithms

**Figure 2:**
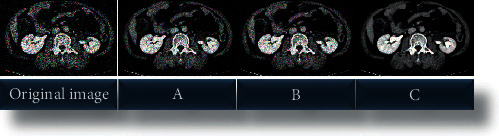
Processing effects of three algorithms. (a), (b), and (c) show the processing effects of MFD algorithm, OWD algorithm, and IWT algorithm, respectively.

The algorithm used in this work (IWT algorithm) was compared with the mean filter denoising (MFD) algorithm [23] and the orthogonal wavelet denoising (OWD) algorithm. Figure 1 shows the comparison of MSE and SNR of CT images processed by three algorithms. Among them, the MSE values of the algorithm adopted in this work, the MFD algorithm, and the OWD algorithm were 40.0781, 45.2891, and 59.2123, respectively; the SNR values were 20.0122, 18.2311, and 15.7812, respectively. It can be found that the denoising effect of the algorithm used in this work was better than other algorithms (*P* < 0.05).  Figure 2 shows the processing effect of the CT images of the three algorithms. It can be observed that the CT image processed by the algorithm used in this study showed a higher definition.

### 3.3. Anthropometric Evaluation Results

**Figure 3:**
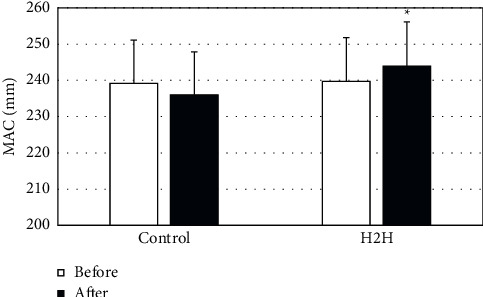
Comparison on MAC before and after nursing. *∗P* < 0.05 indicates the difference was statistically significant, compared with “H2H group before nursing.”

**Figure 4:**
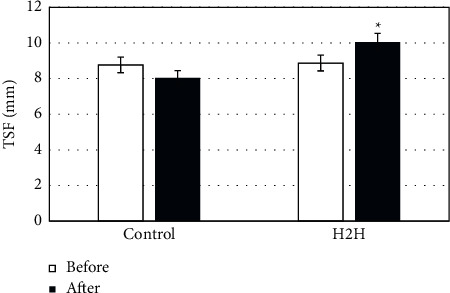
Comparison on TSF before and after nursing. *∗P* < 0.05 indicates the difference was statistically significant, compared with “H2H group before nursing.”

Figure 3 shows that the MAC of the patients in the H2H group after nutritional care was significantly improved compared with that before the nursing ((239.77 ± 18.24 vs 243.94 ± 18.72) mm) (*P* < 0.05), and the nursing effect was better than that of the control group.  Figure 4 shows that TSF after nursing was improved compared with before nursing ((8.87 ± 2.74 vs 10.04 ± 2.90) mm) (*P* < 0.05), and the nursing effect was better than that of the control group.

### 3.4. Laboratory Biochemical Test Results

Table 1 lists the comparison of the laboratory biochemical indexes (ALB, PAB, Hb, BUN, and SCr) of the two groups of patients before nutritional nursing and 6 months after nursing. It revealed that the various biochemical indicators of the control group patients had a certain improvement, and there was no significant difference in comparison (*P* > 0.05). ALB, PAB, Hb, and BUN of patients in the H2H group after nursing for 6 months were significantly improved compared with those before nursing (*P* < 0.05).

### 3.5. Questionnaire Evaluation Results

**Figure 5:**
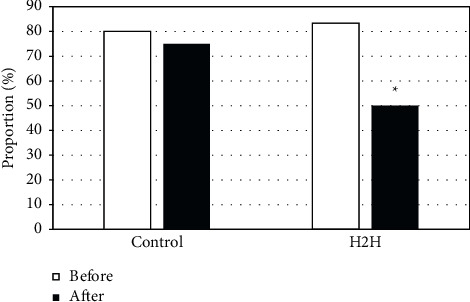
Comparison on NRS 2002 evaluation results. *∗P* < 0.05 indicates the difference was statistically significant, compared with “H2H group before nursing.”

Figure 5 shows the statistical comparison of the NRS 2002 evaluation results of the two groups of patients before and after nursing. In the control group, 48 patients (80%) had an NRS 2002 score ≥3 before nursing and 45 patients (75%) after nursing, so the comparison was not significantly different (*P* > 0.05). In the H2H group, there were 50 patients (83.33%) with NRS 2002 score ≥3 before nursing and 30 patients (50%) after nursing, so there was a significant difference in comparison (*P* < 0.05).

Table 2 lists the scoring results of the four dimensions of the SF-36 summary table of the two groups of patients: mental function, physiological function, social relationship, and treatment status. Calculation and analysis showed there was no significant difference between the control group and H2H group in the total score of health quality assessment before nursing (*P* > 0.05); while the total score of health quality assessment in the H2H group after nursing was much higher than that of the control group (*P* < 0.05).

Table 3 lists the statistics of the survey results of the satisfaction of the two groups of patients with the nursing process. In the control group, 43 cases (71.67%) were satisfied with the nutritional nursing model and 60 cases (100%) were satisfied in the H2H group. There was a significant difference (*P* < 0.05).

### 3.6. CT Examination Results

**Figure 6:**
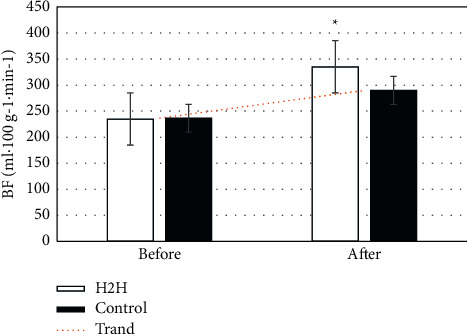
Changes in renal cortex BF before and after nursing.*∗P* < 0.05 indicates the difference was statistically significant, compared with the control group.

**Figure 7:**
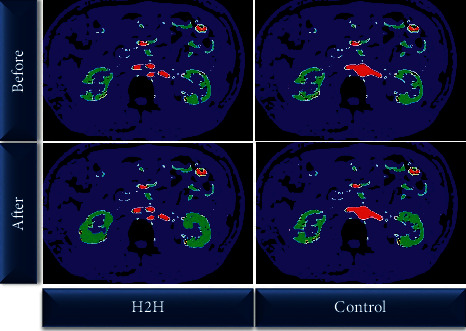
Image display of low-dose CT perfusion imaging.

Figure 6 shows the changes in renal cortex BF of the kidneys of the two groups of patients before and after 6 months of nursing. It can be observed that before nursing, the BF of the two groups was 236.53 ± 44.03 mL·100 g^−1^·min^−1^ in the control group and 234.89 ± 45.11 mL·100 g^−1^·min^−1^ in the H2H group, showing no significant difference (*P* > 0.05). After 6 months of nursing, the BF of the control group was 289.90 ± 53.91 mL 100 g^−1^·min^−1^, while that in the H2H group was 335.12 ± 52.74 mL·100 g^−1^·min^−1^. The BF of the two groups of patients after 6 months of nursing was higher than before, and the H2H group was significantly higher than that of the control group (*P* < 0.05).  Figure 7 shows the display of CT images before and after nursing.

## 4. Discussion

In this study, the “Internet + *H*2H” nutritional nursing model was compared with the conventional nursing model, and the anthropometric indicators, laboratory biochemical indicators, NRS 2002, SF-36 short form, satisfaction survey form, and 320-slice volume CT low-dose perfusion imaging technology were adopted to evaluate the effect of nursing. Malnutrition in CKD patients can cause abnormalities in the muscle tissue and blood biochemical indicators [24]. In this study, the MAC, TSF values, ALB, and PAB of the two groups of nursing provided support for this view. Moreover, the anthropometric indicators showed that the MAC and TSF values of patients in the H2H group were greatly improved after nursing; laboratory biochemical indicators showed that ALB, PAB, Hb, and BUN in the H2H group improved after nutritional nursing. The above results suggest that the nutritional nursing effect of patients in the H2H group is better, which is consistent with the research conclusion of Al-Kalaldeh et al. [25].

Some studies have proposed that the “Internet + *H*2H″ nutritional nursing model is not limited by time and space and has more continuity and pertinence in patients' nursing, which is lacking in traditional nursing methods [26]. The NRS 2002 evaluation results in this study showed that the proportion of patients in the H2H group who were malnourished or at risk of nutrition improved significantly after nursing. Another research suggests that the Internet can better realize effective interaction and resource sharing between nursing staff and patients, give patients individualized guidance, correct patients' unhealthy conditions timely and effectively, improve patients' physical and psychological conditions, and enhance patients' cure confidence [27, 28]. In this study, the SF-36 short form was adopted to evaluate, and it was found that the scores of mental function, physiological function, social relationship, and treatment status of patients in the H2H group were better than those of the control group after 6 months of nursing, so the patients in H2H group were more satisfied with the nursing. In addition, the study also used 320-slice volume CT low-dose perfusion imaging to detect the renal function of patients before and after nursing. There was a significant difference in renal cortex BF between the two groups before and after nursing (*P* > 0.05), and the BE of the H2H group was significantly higher than that of the control group after treatment (335.12 ± 52.74 mL·100 g^−1^·min^−1^ vs 289.90 ± 53.91 mL·100g^−1^·min^−1^) (*P* < 0.05). It was suggested that the prognosis of the patient's disease was related to the nutritional status of the body, and the application effect of the “Internet + *H*2H″ nutritional nursing model was better. Such results are in line with the research conclusions drawn by Lukaski et al. (2017) [29, 30]. The H2H nutritional care model is not only suitable for kidney care but also still applicable in neonatal care [31] and respiratory system disease care [32], and the application effect is good.

In order to improve the accuracy of the detection results, the IWT algorithm was used to denoise the CT image and analyze its performance. The results showed that compared with the MFD algorithm and the OWD algorithm, the MSE and SNR values of the denoising algorithm in this study were better. Hsieh et al. (2018) [30] improved the wavelet transform algorithm and used it for CT image processing and found that this method had higher SNR, better structural similarity index, and lower relative error.

## 5. Conclusion

In this study, the “Internet + *H*2H″ nutritional nursing model was adopted to perform nutritional nursing for patients with CKD in stages 3-5, and the CT images based on the IWT algorithm and other evaluations were adopted to evaluate their nursing effects, so as to compare with traditional nursing methods. The results showed that the Internet + *H*2H “nutritional nursing model was more individual and can better improve the physical quality of patients with stages 3-5, mental state of patients, and prognosis of the disease. In addition, the IWT algorithm had better results in the processing of 320-slice volume CT low-dose perfusion imaging and was worthy of clinical application. However, this study did not separately conduct targeted studies on patients in phases 3 to 5, and the records of the research indicators were not detailed enough, which are needed to be strengthened in subsequent studies. However, the overall results of the study showed that Internet technology had achieved an important position in the field, and the “Internet + *H*2H″ nutritional nursing model would also be widely recognized by patients and doctors in the application of nutritional nursing for patients in the future.

## Figures and Tables

**Table 1:** Comparison of the laboratory biochemical indexes of the two groups of patients before nutritional nursing and 6 months after nursing.

Biochemical indexes	Control group (*n* = 60)		H2H group (*n* = 60)	
	Before nursing	Six months after nursing	Before nursing	Six months after nursing
ALB (g/L)	34.32 ± 10.21	36.32 ± 9.89	35.22 ± 4.98	45.32 ± 4.21^*∗*^
PAB (mg/L)	297.74 ± 71.82	307.12 ± 72.81	289.94 ± 72.99	341.79 ± 74.45^*∗*^
Hb (g/L)	98.61 ± 24.87	103.61 ± 24.87	97.62 ± 24.87	110.65 ± 28.83^*∗*^
BUN (mmol/L)	15.28 ± 12.34	15.40 ± 11.68	15.74 ± 9.87	11.06 ± 5.69^*∗*^
SCr (*μ*mol/L)	548.95 ± 115.50	547.98 ± 101.87	551.70 ± 115.28	543.59 ± 115.33^*∗*^

^*∗*^ *P* < 0.05 indicates the difference was statistically significant, compared with “H2H group before nursing.”

**Table 2:** Scoring results of the four dimensions of the SF-36 summary table of two groups of patients.

Biochemical indexes	Control group (*n* = 60)		H2H group (*n* = 60)	
	Before nursing	Six months after nursing	Before nursing	Six months after nursing
Mental function	36.45 ± 5.01	37.89 ± 4.29	35.98 ± 4.38	49.32 ± 3.21*∗*
Physiological function	18.32 ± 5.21	18.82 ± 4.81	18.22 ± 5.98	27.33 ± 4.67*∗*
Social relationship	16.33 ± 3.51	17.02 ± 3.67	15.23 ± 3.34	20.32 ± 1.01*∗*
Treatment status	11.21 ± 4.25	12.11 ± 3.49	10.87 ± 4.98	19.24 ± 1.89*∗*

*∗P* < 0.05 indicates the difference was statistically significant, compared with “H2H group before nursing.”

**Table 3:** Comparison on the satisfaction of the two groups of patients.

	Very satisfied	Satisfied	Not satisfied	Percentage
Control group (*n* = 60)	26	17	17	71.67
H2H group (*n* = 60)	42	18	0	100*∗*

*∗P* < 0.05 indicates the difference was statistically significant, compared with the control group.

## Data Availability

The data used to support the findings of this study are available from the corresponding author upon request.
